# Using Odd-Alkanes as a Carbon Source to Increase the Content of Nutritionally Important Fatty Acids in* Candida krusei, Trichosporon cutaneum, *and* Yarrowia lipolytica*

**DOI:** 10.1155/2017/8195329

**Published:** 2017-10-10

**Authors:** Olga Matatkova, Lucia Gharwalova, Michal Zimola, Tomas Rezanka, Jan Masak, Irena Kolouchova

**Affiliations:** ^1^Department of Biotechnology, University of Chemistry and Technology, Prague, Technicka 5, 166 28 Prague, Czech Republic; ^2^Institute of Microbiology, Academy of Sciences of the Czech Republic, Videnska 1083, 142 20 Prague, Czech Republic

## Abstract

We investigated the possibility of utilizing unusual carbon sources by three yeast strains:* Candida krusei* DBM 2136,* Trichosporon cutaneum* CCY 30-5-10, and* Yarrowia lipolytica* CCY 30-26-36. These strains are characterized by high biomass yield, ability to accumulate high amounts of lipids, and their potential as producers of dietetically important fatty acids. The aim of this work was the production of nutritionally important fatty acids by utilization of n-alkanes with an odd number of carbon atoms, alone and in combination with glucose and subsequent analysis of microbial lipids accumulation and fatty acid profile. All three yeast strains were able to grow and produce high amounts of the fatty acids of interest.* Yarrowia lipolytica *was found as the most suitable strain for the growth on n-alkanes (n-pentadecane and n-heptadecane) as the only source of carbon. The addition of biosurfactants rhamnolipids into the cultivation increased the ratio of heptadecenoic acid (up to 17.9% of total FAs in* Y. lipolytica CCY 30-26-36*, 14.9% in* T. cutaneum CCY 30-5-10, *and 17.5% in* C. krusei DBM 2136*) and the total biomass yield. The results show that, by manipulation of the initial cultivation conditions, the ratio of important fatty acids may be increased.

## 1. Introduction

Yeasts have great potential in biotechnology due to their easy cultivation and high growth rate [1, 2]. The cultivation of yeast cells takes place in cultivation media rich in elements necessary for cell growth [3, 4]. The main element for yeasts, which are heterotrophic organisms, is carbon. Traditionally, glucose is used as a main source of carbon; however, its high price makes it economically unsuitable for large-scale cultivation. Therefore, it is desirable to look for cheaper and less traditional carbon sources, for example, waste materials. At the present time, great attention is paid to the possibility of utilization of alkanes as carbon sources by microorganisms, since these substances represent pollutants to our environment and are obtainable as by-products from some industries.

Alkanes are petroleum fractions, which are present in high proportions but must be removed during the production process of fuels due to their unfavorable melting points. Alkanes can also be found in wax layers of plants and animals to reduce their loss of water by evaporation [5]. They may be linear (n-alkanes) or branched (iso-alkanes) and are, because of their chemistry, only slightly soluble in water. The fact that they are nonpolar and inert organic compounds [6] represents a challenge in their use as substrates for microorganisms. The ability of microorganisms to utilize hydrocarbons as sole carbon source was first described in 1895 [7]. However, the bioavailability of hydrophobic organic compounds to microorganisms was found as a limiting step during the biodegradation process [8].

Representatives of bacteria, yeast, and fungi were proven able to be grown solely on petroleum hydrocarbons. Yeast strains especially are possible to utilize for bioremediation of petroleum contaminated industrial wastewaters [9]. Yeasts capable of degrading hydrocarbons include* Y. lipolytica*,* C. tropicalis*,* C. albicans*,* T. asahii,* and* D. hansenii*. The yeast* Y. lipolytica* has been extensively studied due to its high efficiency in degradation of hydrophobic substrates such as alkanes, triacylglycerides, and fatty acids [10].* Y. lipolytica* and its catabolism of hydrocarbons belong to the most studied strains [11].* C. maltosa* strains were reported to utilize n-hexadecane faster than glucose [5, 12].

Main lipids synthesized in yeast are triglycerides (consisting of saturated, mono-, or polyunsaturated fatty acids). The fatty acids (FAs) originating in yeasts can be used to produce dietetic products, which are essential for human nutrition. The FAs are part of microbial lipids and can therefore be obtained in larger quantities by modifying the culture conditions that induce an increase in microbial lipid production [13] or by synthesis of specific lipids [14]. The FAs nutritional value is determined by the fatty acid type, especially by the number of carbon atom and the number and position of unsaturated bonds. Several types of fatty acids are nowadays investigated with respect to human health and their requirements in the diet [15]. It was demonstrated that pentadecenoic (CH_3_(CH_2_)_13_COOH) and heptadecenoic (CH_3_(CH_2_)_15_COOH) acid have a positive effect on the human health and contribute to reduction of the risk of developing multiple sclerosis [16]. Heptadecenoic acid functions as an anti-inflammatory and edema-inhibiting compound, and it is also active on psoriasis, allergies, and autoimmune diseases, especially in prophylaxis [17], and it also inhibits plant diseases such as powdery mildew [18, 19]. Monounsaturated fatty acids are preferred substrates for acyl-CoA-cholesterol acyltransferase, which catalyzes the esterification of hepatic free cholesterol to an inert cholesterol ester pool. This in turn reduces the putative regulatory pool of intracellular free cholesterol, increasing low-density lipoprotein (LDL) receptor activity and subsequently decreasing circulating cholesterol concentrations in humans [20]. Odd-numbered FAs such as heptadecenoic acid in nutrition are usually obtained from mammalian milk and fat [21] or seeds of plants of the Malvaceae family [22, 23]. Some types of higher basidiomycete fungi, such as* Ganoderma applanatum,* are also known producers of heptadecenoic acid [24]. Palmitoleic acid-rich diets have also been reported to improve circulating lipid profile, resulting in reduced total and LDL cholesterol [20]. Linoleic acid is a polyunsaturated n-6 fatty acid that participates in the fat metabolism and also has been also proven to have positive effects in the treatment of many diseases [25]. Furthermore, heptadecenoic acid has a very suitable cetane number, which was calculated according to Ramírez-Verduzco et al. [26] and reaches values greater than 57, so it would fit as an excellent additive into biodiesel.

While studying the utilization of various kinds of hydrocarbons, it has been found that some bacteria and yeasts can directly transform the n-alkanes to fatty acids without their degradation to acetate [5, 27]. Microorganisms growing on n-alkanes with a length of 15 to 18 carbons incorporated these hydrocarbons directly into the cellular fatty acids [27]. The use of n-alkanes with a length of chain 15 carbons and 17 may could thus increase the content of C15 and C17 fatty acids, which could be further isolated and purified from the biomass [28].

Surfactants are agents, which reduce the interfacial tension between two liquids or between a liquid and a solid [29, 30]. The ability to produce biosurfactants was described in bacteria, yeasts, and fungi [31]. The collection of data on the structure and production of biosurfactants is important for the enhancement of many industry fields, bioremediation technologies, oil industry, agriculture, cosmetics, therapeutics, and microbial enzymes production [32–35], with special focus on the yeast species belonging to* Candida, Rhodotorula,* and* Yarrowia *[36, 37]. Biosurfactants display some advantageous properties, low surface tension, low critical micelle concentration (CMC), high affinity for hydrophobic organic substrates, and high emulsifying activities [38]. Biosurfactants are also often easily biodegradable and environmentally safe [39].* Candida* and* Yarrowia* strains when grown on hydrocarbons were found to produce sophorolipids, mannosylerythritol lipids, carbohydrate-protein-lipid complexes, carbohydrate-protein complexes, or fatty acids [40–42].* Y. lipolytica* produced biosurfactant during cultivation both on hydrophobic substrates and glucose as a carbon sources [43]. Yeast cells that do not produce their own surfactants need their addition in order to utilize alkanes more efficiently. Rhamnolipids rank among the best-known and most efficient biosurfactants. These compounds are produced by the bacteria* Pseudomonas aeruginosa* and are mainly used because of their low toxicity and ease of biodegradability. Several studies have showed that rhamnolipids may be potentially useful in the degradation and solubilization of alkanes [44, 45] and in the production of citric acid production from sunflower oil [46].

In this study, we investigated the ability of three yeast strains (*C. krusei* DBM 2136;* T. cutaneum* CCY 30-5-10;* Y. lipolytica* CCY 30-26-36) to grow on media containing either solely odd n-alkanes or odd n-alkanes with glucose as a source of carbon and energy. We also tested whether an addition of rhamnolipids could improve the utilization of n-alkanes and change the lipid yield and fatty acid profile of our yeast strains.

## 2. Materials and Methods

### 2.1. Microorganisms

The yeast strains used in the present study were* C. krusei *DBM 2136;* T. cutaneum* CCY 30-5-10;* Y. lipolytica* CCY 30-26-36 supplied by Culture Collection of Yeast, Institute of Chemistry, Slovak Academy of Sciences, Bratislava, and by Collection of Yeasts and Industrial Microorganisms of University of Chemistry and Technology, Prague. For long storage, the stock cultures were maintained in 20% glycerol at −70°C.

### 2.2. Cultivation Conditions

**Figure 1:**
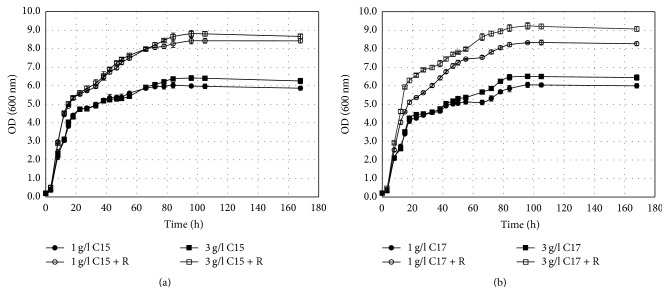
Growth curves* Y. lipolytica CCY 30-26-36* on glucose with pentadecane (a) and glucose with heptadecane (b) as a carbon source (R = addition of rhamnolipid).

**Figure 2:**
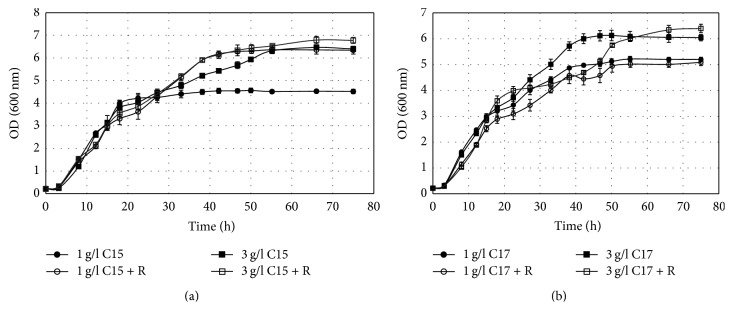
Growth curves of* Y. lipolytica CCY 30-26-36* on pentadecane (a) and heptadecane (b) as a sole carbon source (R = addition of rhamnolipid).

The precultures of yeast strains were cultivated in 100 mL of complex yeast extract peptone dextrose (YPD) medium (20 g/L peptone (Cat.# P6838, Sigma-Aldrich, Darmstadt, Germany), 10 g/L yeast extract (Cat.# Y1625, Sigma-Aldrich, Darmstadt, Germany), 20 g/L glucose (Cat.# D9434, Sigma-Aldrich, Darmstadt, Germany) initial pH 6, sterilized at 121°C for 20 min) in 500 mL Erlenmeyer flasks on a rotary shaker at 150 rpm at 28°C to the late exponential growth phase (according to Kolouchova et al. [3]). For lipid production, 200 mL of mineral medium in 500 mL Erlenmeyer flasks was inoculated with 10 mL of preculture to a final concentration of OD_600_ 0.2 and incubated on a rotary shaker at 150 rpm and 28°C to the stationary phase. Mineral medium composition was (g/L) KH_2_PO_4_, 1.7; Na_2_HPO_4_·2H_2_O, 0.75; (NH_4_)_2_SO_4_, 4.00; MgCl_2_·6H_2_O, 0.34; trace element solution 1 mL (MnCl_2_·4H_2_O, 0.34; CaCl_2_·2H_2_O, 0.26; FeSO_4_·7H_2_O, 2.84; NaMoO_4_·2H_2_O, 0.34), pH 6, sterilized at 121°C for 20 min. In experiment with glucose, concentration 20 g/L was employed. All experiments were performed in triplicate (independent cultivations; *n* = 3, standard deviations were included in Figures 1 and 2 and Table 1S in Supplementary Material available online at https://doi.org/10.1155/2017/8195329 where appropriate).

Yeast strains were cultivated with n-pentadecane (≥99%, Cat.# P3406, Sigma-Aldrich, Darmstadt, Germany) or n-heptadecane (≥99%, Cat.# 128503, Sigma-Aldrich, Darmstadt, Germany) added in concentration 1 g/L or 3 g/L. These concentrations were based upon previous unpublished preliminary laboratory studies. All cultivations were performed with or without the supplement of rhamnolipids. Rhamnolipid obtained from* Pseudomonas aeruginosa* DBM 3775 was produced, purified, and characterized as described previously [47]. Briefly, after cultivation of* P. aeruginosa*, the rhamnolipids were isolated from the supernatant by acidic precipitation and purified by extraction and TLC. The composition of the purified rhamnolipid extract was determined by tandem mass spectrometry and was found to consist of 27 congeners from all four rhamnolipid classes (RhaFA, RhaFAFA, RhaRhaFA, and RhaRhaFAFA), which contained five types of fatty acids (C8, C10, C10:1, C12, and C12:1). Rhamnolipids containing decanoic acid formed the major component (60%). For the experiments, the purified rhamnolipid mixture was used. Rhamnolipids were added in critical micellar concentration 56 mg/L.

After cultivation, the cells were centrifuged (10 min, 9000*g*, Hettich Rotina 380, Germany) and washed two times according to Chrzanowski et al. [7]. Cell mass was frozen at −70°C and lyophilized. Biomass yield was determined as dry cell weight.

### 2.3. Lipid Extraction

Lyophilized yeast biomass was mixed with 2 mL of 0.1 mol/L Na_2_CO_3_ (≥99%, Cat.# 451614, Sigma-Aldrich, Darmstadt, Germany) and the mixture was briefly ground with ballotini glass beads (diameter 0.2 mm) in a mortar, overlaid with liquid nitrogen, and ground again. This process was repeated 3 times and 50 mL of 0.1 mol/L Na_2_CO_3_ was finally added. The lipids were extracted with chloroform-methanol mixture according to Bligh and Dyer [48]. The sample was centrifuged and the lower phase was evaporated to dryness and the lipid dry weight was determined.

### 2.4. Analysis of Fatty Acid Methyl Esters

The total lipids (~5 mg) were saponified overnight in 10% KOH-MeOH at room temperature. The fatty acid fraction obtained from the saponification was partitioned between alkali solution (pH 9) and diethyl-ether to remove basic and neutral components. The aqueous phase containing fatty acids was acidified to pH 2 and extracted with hexane. The fatty acid fraction was methylated using BF_3_/MeOH (14% solution of BF_3_, Cat.# B1252, Sigma-Aldrich, Darmstadt, Germany).

Gas chromatography-mass spectrometry of FAMEs (fatty acid methyl esters) was done on a GC-MS system consisting of Varian 450 GC (Varian BVm Middleburg, The Netherlands), Varian 240-MS ion trap detector with electron ionization (EI), and CombiPal autosampler (CTC, USA) equipped with split/splitless injector. A SP-2380 column (Supelco) (100 m, 0.25 mm ID, 0.20 *µ*m film thickness) was used for separation. The temperature program started at 60°C and was held for 1 min in splitless mode. Then the splitter was opened and the oven heated to 160°C at the rate of 25°C min^−1^. The second temperature ramp was up to 220°C at a rate of 1.0°C min^−1^, this temperature being maintained for 10 min. The solvent delay time was set to 8 min. The transfer line temperature was set to 280°C. Mass spectra were recorded at 3 scans s^−1^ under electron ionization at 70 eV, mass range* m*/*z* 500–600. FAMEs were identified according to their mass spectra [49, 50] and using mixture of chemical standards obtained from Sigma-Aldrich (FAME Mix C4-C24, Cat.# 18919- 1AMP).

### 2.5. Statistical Analysis

Statistical analysis was performed with SigmaStat 3.5 (USA). The statistical significance of differences in mean values of the different measured parameters was calculated by one-way ANOVAs and compared with Tukey's test at the 5% level of probability.

## 3. Results and Discussion

### 3.1. Effect of Odd n-Alkanes on Growth and Lipid Content of Yeast Strains

There is only a limited amount of literature on the topic of n-alkanes as sole carbon sources for yeast lipid production [51, 52] and the published results mostly describe only few species (usually* C. maltosa* or* Y. lipolytica*). We have performed preliminary screening of cultivation on pentadecane and heptadecane as sole carbon sources and in combination with glucose in three yeast strains,* C. krusei DBM 2136*,* T. cutaneum CCY 30-5-10,* and* Y. lipolytica CCY 30-26-36. *n-Alkanes are only marginally soluble in water; their solubility decreases with the length of the carbon chain [53]. Only some microorganisms are able to utilize such compounds, usually by producing biosurfactants [54, 55]. In non-biosurfactant producing strains, the addition of exogenous surfactants may improve the bioavailability of the hydrophobic substrate [56].

All three yeasts (*C. krusei* DBM 2136;* T. cutaneum* CCY 30-5-10;* Y. lipolytica* CCY 30-26-36) preferred media containing both glucose and alkanes to media with alkanes alone (Figure 1 and Fig. 1S and Fig. 3S). The highest growth on n-alkanes (n-pentadecane and n-heptadecane) was observed in* Y. lipolytica CCY 30-26-36* and the lowest in* T. cutaneum CCY 30-5-10 *(Figure 2 and Fig 2S and Fig. 4S and Table 1). In all cases, the addition of low concentration of rhamnolipids had a positive effect on biomass yield; 10–30% increase was observed for* C. krusei DBM 2136 *and* Y. lipolytica CCY 30-26-36*, but up to several-fold increase in* T. cutaneum CCY 30-5-10 *was observed. Significant increase of biomass yield in higher concentration of alkanes was observed only in* T. cutaneum CCY 30-5-10,* where the increase from 1 g/L to 3 g/L of alkanes led to three-times the increase in biomass yield.

In microorganisms, de novo lipid synthesis and ex novo lipid accumulation are the two main pathways of lipid synthesis [2]. De novo lipid accumulation is achieved by cultivation on hydrophilic substrates, saccharides or short chain FAs [57]. Hydrophobic substrates utilization leads to ex novo lipid accumulation. The substrate, cultivation conditions, inoculum, temperature, and other factors directly influence the microbial lipid content and composition [2, 57]. In our work, the cultivation temperature was 28°C and initial pH 6, based on our previous results [3]. Cultivation was carried out until late exponential phase. As can be seen from Table 1, the lipid content was strain- and carbon source-dependent. Higher lipid content (30%) was achieved in the cultivation with alkanes and glucose than in cultivation with alkane as sole carbon source. The influence of rhamnolipid was also specific to each yeast strain.* Y. lipolytica CCY 30-26-36 *showed a 15% increase of total lipids in the presence of rhamnolipid in cultivation on n-alkane and glucose and up to 30% in cultivation solely on n-alkane. Białas et al. [46] reported that the addition of rhamnolipids during cultivation of* Y. lipolytica CCY 30-26-36 *grown on hydrophobic substrate contributed to the solubilization of the corresponding substrate and a stable emulsion was formed. Rhamnolipids interacted with* Y. lipolytica* cells by significantly decreasing their hydrophobicity, which may have contributed to the reduction of substrate uptake.

The addition of rhamnolipid to* C. krusei DBM 2136 *increased the content of lipids by 15% when pentadecane was utilized and decreased by 10% with heptadecane. A similar trend was found in* T. cutaneum* in the presence of glucose and n-alkane. In this case, the lipid content increased in the presence of rhamnolipid and decreased by 20% in cultivations solely with n-alkane. These trends correspond with the results obtained on hydrophobic substrates [3, 58] and it is therefore possible to suggest a similar application of n-alkanes as inexpensive substrates for the biotransformation into microbial lipids.

### 3.2. Fatty Acids Composition of Lipids

The influence of n-alkanes with odd number of carbon atoms on lipid composition of three yeast strains was determined by GC-MS. Table 1 shows the ratio of saturated and unsaturated fatty acids. The lowest content of unsaturated fatty acids was observed in* C. krusei DBM 2136 *(67–76%)*; T. cutaneum CCY 30-5-10 *and* Y. lipolytica CCY 30-26-36 *contained about 80% of unsaturated FAs. This work is the first to our knowledge to study these three yeast strains cultivation on odd-numbered alkanes with the addition of rhamnolipids. Under these unusual cultivation conditions, the major FAs were palmitic acid (16:0), oleic acid (18:1), and linoleic acid (18:2), similarly to reported data in traditional cultivations [2, 57–59]. This fatty acid composition is similar to plant oils that are used as substrates for biofuel production [57, 60, 61].

The synthesis of FAs is regulated by the production of intermediates (acyl-CoA). Odd-numbered FAs are synthetized usually from precursors with odd numbers of carbon atoms (e.g., propionyl-CoA) or are formed by chain shortening of even-numbered FAs.

The content of odd FAs was majorly influenced by the cultivation on n-alkane as the sole carbon source with the addition of rhamnolipid. In the presence of pentadecane, a significant increase of C15 FAs is observed and, in the presence of heptadecane, the corresponding content of C17 FAs increases (Table 1, Table 1S with standard deviations). The content of odd FAs is shown in Table 1 (Table 1S with standard deviations) and was up to 44% of the total lipids (*Y. lipolytica CCY 30-26-36*, 3 g/L pentadecane, with rhamnolipid). The overall effect of the presence of n-alkane as the sole carbon source with the addition of rhamnolipid leads to an increase of the FAs content up to 20–30% of the total lipids. The highest content of heptadecenoic acid was found in* C. krusei DBM 2136 *and* Y. lipolytica CCY 30-26-36 *(17% from total FAs), which is significantly more than results obtained in other studies, when yeasts grew on glucose or even short chain fatty acids [3, 62, 63]. Other nutritionally important FAs comprise palmitoleic and linoleic acid. The highest content of palmitoleic acid was 14% in* Y. lipolytica CCY 30-26-36* and 10% in* C. krusei DBM 2136. T. cutaneum CCY 30-5-10 *was found exceptional in the relative linoleic acid content, surpassing 50% of the total FAs. In* C. krusei DBM 2136 *linoleic acid formed 30–40% of total FAs. The yields of the nutritionally interesting FAs (17:1, 16:1, 18:2) are shown in Table 1. The highest yield of heptadecenoic acid was observed in* C. krusei DBM 2136 *(96 mg/L) in cultivation on 3 g/L heptadecane with glucose and rhamnolipid. This value is similar to results reported previously by Řezanka et al. [4] in cultivation on propionic acid. The second highest yield (83 mg/L) was in* Y. lipolytica CCY 30-26-36 *(3 g/L heptadecane, with rhamnolipid). Palmitoleic acid yield was the highest (100 mg/L) when* C. krusei DBM 2136 *or* Y. lipolytica CCY 30-26-36 *were cultivated on n-alkanes with glucose. These cultivation conditions lead to the 300 mg/L yield of linoleic acid in* C. krusei DBM 2136 *and* T. cutaneum CCY 30-5-10. T. cutaneum CCY 30-5-10 *when cultivated on 3 g/L of pentadecane (with glucose and rhamnolipid) reached the yield of linoleic acid 1200 mg/L.

When the biotechnological application of microbial biomass is considered, the content of microbial lipids, as well as biomass yield, is important. However, it is also possible to prioritize the financial costs of the substrate or the FAs profile.

Even though unusual substrates were employed, we have shown that in* C. krusei DBM 2136 *the content of palmitic, oleic, and linoleic acid (up to 60% of total lipids) is similar to results obtained on more usual carbon sources, such as glucose [64, 65]. Oleic acid was the most represented FA in* Y. lipolytica CCY 30-26-36 *(approx. 50%),* T. cutaneum CCY 30-5-10 *showed a high content of linoleic acid, more than 50% of total FAs, under all cultivation conditions.

## 4. Conclusions

All the studied yeast strains (*C. krusei* DBM 2136;* T. cutaneum* CCY 30-5-10;* Y. lipolytica* CCY 30-26-36) were able to grow on n- alkanes with odd number of carbon atoms, albeit to a different degree.* Y. lipolytica CCY 30-26-36 *displayed the highest growth characteristics. High ratio of unsaturated fatty acids was found in all three yeast strains under all cultivation conditions, mostly over 80% of total FAs. The highest lipid to biomass ratio was observed in* T. cutaneum CCY 30-5-10. Y. lipolytica CCY 30-26-36 *may be used for cultivation due to its ability to grow on unusual substrates and concomitantly produce high amount of nutritionally important FAs (over 85% of oleic acid, palmitoleic acid, and linoleic acid of the total lipid content). The application of rhamnolipid might be means to the modulation of the proportion of odd- and even-numbered FAs. In our future experiments, we would like optimize the lipid production with selected yeast and n- alkanes in laboratory bioreactors of 1 and 5 L volume.

## Supplementary Material

Effect of different substrates (pentadecane C15; heptadecane C17 in concentration 1 g/L or 3 g/L) with or without presence of rhamnolipid (R) or glucose (G) on biomass yield (X), total lipid production (L) and lipid yield (L/X), and the proportion of saturated, unsaturated, odd, and even fatty acids by three yeast strains. The highest odd fatty acid content is shown in bold. The standard deviations are included.

## Figures and Tables

**Table 1:** Effect of different substrates (pentadecane C15; heptadecane C17 in concentration 1 g/L or 3 g/L) with or without presence of rhamnolipid (R) or glucose (G) on biomass yield (X), total lipid production (L) and lipid yield (L/X), and the proportion of saturated, unsaturated, odd, and even fatty acids by three yeast strains. The highest odd fatty acid content is shown in bold.

*Candida krusei*																		
Substrate	Characteristic	L/X (% w/w)	L (g/L)	X (g/L)	Unsat FA	Sat FA	Odd FA	Even FA	Odd /even ratio	15:0	15:1	16:0	16:1	17:0	17:1	18:0	18:1	18:2

G+C15	1C15	17.40	0.86	4.94	66.9	33.1	1.6	98.4	0.016	0.6	0.5	21.1	11.0	0.0	0.5	11.4	15.3	39.6
G+C15	R1C15	20.70	1.19	5.75	73.7	26.3	6.1	93.9	0.065	1.3	2.3	16.6	9.8	0.9	1.6	7.5	33.5	26.5
G+C15	3C15	16.05	1.01	6.31	67.5	32.5	1.0	99.0	0.010	0.6	0.0	24.2	11.1	0.2	0.2	7.5	13.3	42.9
G+C15	R3C15	20.40	1.37	6.69	73.4	26.6	8.1	91.9	0.088	1.1	5.0	16.4	9.8	1.1	0.9	8.0	35.7	22.0
G+C17	1C17	20.40	1.12	5.49	72.3	27.7	2.2	97.8	0.022	0.4	0.6	21.4	11.0	0.4	0.8	5.5	17.5	42.4
G+C17	R1C17	21.30	1.29	6.05	73.8	26.2	10.6	89.4	0.119	0.9	1.2	14.6	9.2	4.2	4.3	6.5	38.2	20.9
G+C17	3C17	23.70	1.16	4.88	73.5	26.5	3.6	96.4	0.037	0.5	2.0	19.9	10.7	0.4	0.7	5.7	18.3	41.8
G+C17	R3C17	21.15	1.40	6.60	73.1	26.9	13.4	86.6	0.155	0.6	0.8	15.9	8.9	5.1	6.9	5.3	32.1	24.4
C15	1C15	13.65	0.07	0.50	73.6	26.4	5.5	94.5	0.058	1.2	1.7	16.7	10.0	1.0	1.6	7.5	33.8	26.5
C15	R1C15	14.70	0.09	0.58	69.4	30.6	11.9	88.1	0.135	7.3	2.1	21.2	10.9	1.0	1.5	1.1	15.3	39.6
C15	3C15	13.50	0.08	0.57	73.6	26.4	7.3	92.7	0.079	0.7	4.5	16.6	9.9	1.1	1.0	8.0	35.9	22.3
C15	R3C15	15.15	0.10	0.64	65.4	34.6	18.8	81.2	0.232	11.1	4.6	21.3	9.7	1.4	1.7	0.8	11.7	37.7
C17	1C17	15.30	0.09	0.59	76.4	23.6	5.5	94.5	0.058	0.6	2.9	15.4	9.7	0.7	1.3	6.9	40.3	22.2
C17	R1C17	15.00	0.11	0.73	72.8	27.2	27.8	72.2	0.385	0.7	0.6	16.6	8.5	9.6	16.9	0.3	13.6	33.2
C17	3C17	18.45	0.14	0.78	73.2	26.8	12.5	87.5	0.143	1.0	1.3	16.0	9.0	4.4	5.8	5.4	32.5	24.6
C17	R3C17	16.95	0.14	0.81	70.2	29.8	33.5	66.5	0.504	0.7	0.9	14.5	7.8	14.4	17.5	0.2	13.4	30.6

*Trichosporon cutaneum*																		
Substrate	Characteristic	L/X (% w/w)	L (g/L)	X (g/L)	Unsat FA	Sat FA	Odd FA	Even FA	Odd /even ratio	15:0	15:1	16:0	16:1	17:0	17:1	18:0	18:1	18:2

G+C15	1C15	31.80	0.58	1.83	84.0	16.0	0.9	99.1	0.009	0.2	0.3	13.8	2.5	0.1	0.3	1.9	29.4	51.5
G+C15	R1C15	33.40	1.50	4.49	83.6	16.4	1.3	98.7	0.013	0.3	0.5	14.1	2.6	0.2	0.3	1.8	27.5	52.7
G+C15	3C15	30.90	0.60	1.94	82.9	17.1	1.2	98.8	0.012	0.2	0.4	14.7	2.1	0.2	0.4	2.0	26.7	53.3
G+C15	R3C15	32.80	2.37	7.22	83.6	16.4	1.9	98.1	0.019	0.4	0.8	13.8	2.8	0.3	0.4	1.9	28.4	51.2
G+C17	1C17	32.70	0.76	2.32	83.8	16.2	0.9	99.1	0.009	0.0	0.0	14.2	2.8	0.5	0.4	1.5	28.1	52.5
G+C17	R1C17	31.60	1.44	4.56	82.0	18.0	0.9	99.1	0.009	0.0	0.1	15.7	2.2	0.3	0.5	2.0	28.6	50.6
G+C17	3C17	31.80	0.70	2.19	82.8	17.2	1.0	99.0	0.010	0.0	0.1	15.3	2.9	0.3	0.6	1.6	26.4	52.8
G+C17	R3C17	30.70	1.59	5.19	83.4	16.6	1.4	98.6	0.014	0.0	0.2	14.6	2.3	0.5	0.7	1.5	28.1	52.1
C15	1C15	29.20	0.02	0.07	84.2	15.8	1.4	98.6	0.014	0.4	0.7	14.0	2.2	0.2	0.1	1.2	23.3	57.9

*Trichosporon cutaneum*																		
Substrate	Characteristic	L/X (% w/w)	L (g/L)	X (g/L)	Unsat FA	Sat FA	Odd FA	Even FA	Odd /even ratio	15:0	15:1	16:0	16:1	17:0	17:1	18:0	18:1	18:2

C15	R1C15	23.20	0.04	0.19	77.1	22.9	18.3	81.7	0.224	4.1	7.3	13.9	3.0	3.2	3.7	1.7	20.4	42.7
C15	3C15	28.30	0.02	0.06	83.3	16.7	2.6	97.4	0.027	0.8	1.3	14.2	2.0	0.3	0.2	1.4	24.1	55.7
C15	R3C15	23.10	0.07	0.30	73.8	26.2	27.4	72.6	0.377	7.2	9.8	12.3	2.4	4.9	5.5	1.8	16.8	39.3
C17	1C17	27.60	0.02	0.06	82.7	17.3	1.5	98.5	0.015	0.0	0.1	14.8	2.1	0.8	0.6	1.7	29.1	50.8
C17	R1C17	24.50	0.06	0.25	76.4	23.6	20.0	80.0	0.250	0.9	0.7	14.5	2.5	7.0	11.4	1.2	21.3	40.5
C17	3C17	25.40	0.02	0.07	81.9	18.1	2.3	97.7	0.024	0.1	0.2	15.1	2.0	1.1	0.9	1.8	27.2	51.6
C17	R3C17	23.60	0.03	0.13	75.5	24.5	26.5	73.5	0.361	1.2	0.9	12.5	2.2	9.5	14.9	1.3	17.9	39.6

*Yarrowia lipolytica*																		
Substrate	Characteristic	L/X (% w/w)	L (g/L)	X (g/L)	Unsat FA	Sat FA	Odd FA	Even FA	Odd /even ratio	15:0	15:1	16:0	16:1	17:0	17:1	18:0	18:1	18:2

G+C15	1C15	22.80	0.79	3.46	86.8	13.2	0.6	99.4	0.006	0.1	0.2	12.3	13.2	0.1	0.2	0.7	55.6	17.6
G+C15	R1C15	25.60	1.32	5.15	85.7	14.3	0.5	99.5	0.005	0.0	0.2	13.2	12.9	0.1	0.2	1.0	55.6	16.8
G+C15	3C15	21.90	1.11	5.08	84.9	15.1	0.5	99.5	0.005	0.1	0.2	13.8	11.9	0.0	0.2	1.2	54.7	17.9
G+C15	R3C15	24.80	1.43	5.78	86.1	13.9	0.5	99.5	0.005	0.1	0.3	12.6	13.7	0.0	0.1	1.2	56.8	15.2
G+C17	1C17	21.60	0.89	4.11	85.8	14.2	0.6	99.4	0.006	0.1	0.3	13.0	12.7	0.1	0.1	1.0	54.9	17.8
G+C17	R1C17	24.10	1.26	5.21	84.8	15.2	0.5	99.5	0.005	0.0	0.0	14.0	14.1	0.2	0.3	1.0	53.1	17.3
G+C17	3C17	20.10	0.84	4.19	86.0	14.0	1.0	99.0	0.010	0.2	0.4	12.6	13.5	0.1	0.3	1.1	54.5	17.3
G+C17	R3C17	23.30	1.24	5.31	85.5	14.5	0.4	99.6	0.004	0.0	0.1	13.3	14.0	0.1	0.2	1.1	56.4	14.8
C15	1C15	13.40	0.11	0.80	88.1	11.9	0.7	99.3	0.007	0.2	0.4	10.6	15.7	0.0	0.1	1.1	56.1	15.8
C15	R1C15	17.50	0.16	0.89	69.5	30.5	33.8	66.2	0.511	11.3	14.5	14.2	14.2	4.1	3.9	0.9	28.4	8.5
C15	3C15	13.50	0.37	2.73	87.8	12.2	1.3	98.7	0.013	0.4	0.7	10.8	14.9	0.1	0.1	0.9	52.7	19.4
C15	R3C15	17.20	0.47	2.75	67.4	32.6	**44.5**	55.5	0.802	15.6	19.8	11.7	10.8	4.6	4.5	0.7	24.7	7.6
C17	1C17	12.80	0.10	0.74	87.0	13.0	0.4	99.6	0.004	0.0	0.1	12.1	10.8	0.1	0.2	0.8	60.0	15.9
C17	R1C17	17.20	0.13	0.74	75.5	24.5	25.2	74.8	0.337	0.4	0.2	12.9	13.0	10.4	14.2	0.8	40.9	7.2
C17	3C17	12.40	0.34	2.72	87.1	12.9	0.7	99.3	0.007	0.1	0.2	11.7	12.3	0.1	0.3	1.0	57.5	16.8
C17	R3C17	17.00	0.46	2.73	73.3	26.7	31.3	68.7	0.456	0.5	0.5	13.7	12.5	12.4	17.9	0.1	34.5	7.9
