# Supplementary material for: Using Odd-Alkanes as a Carbon Source to Increase the Content of Nutritionally Important Fatty Acids in Candida krusei, Trichosporon cutaneum, and Yarrowia lipolytica
Source: Int J Anal Chem. 2017 Oct 10;2017:8195329. doi: 10.1155/2017/8195329 (PMC5654280; doi:10.1155/2017/8195329)

Table 1S Effect of different substrates (pentadecane C15; heptadecane C17 in concentration 1 g/L or 3 g/L) with or without presence of rhamnolipid (R) or glucose (G) on biomass yield (X), total lipid production (L) and lipid yield (L/X), the proportion of saturated, unsaturated, odd and even fatty acids by three yeast strains provided with standard deviations

S	CH	L/X (% w/w)	L (g/L)	S.D.	X (g/L)	S.D.	unsat FA	S.D. (%)	sat FA	S.D. (%)	odd FA	S.D. (%)	even FA	S.D. (%)	odd/even ratio	15:0	S.D. (%)	15:1	S.D. (%)	16:0	S.D. (%)	16:1	S.D. (%)	17:0	S.D. (%)	17:1	S.D. (%)	18:0	S.D. (%)	18:1	S.D. (%)	18:2	S.D. (%)
Candida crusei																																			
G+C15	1C15	17.40	0.86	0.04	4.94	0.26	66.9	4.92	33.1	5.10	1.6	5.12	98.4	5.12	0.016	0.6	5.24	0.5	4.86	21.1	4.96	11.0	4.95	0.0	5.17	0.5	4.91	11.4	4.92	15.3	5.21	39.6	4.99
G+C15	R1C15	20.70	1.19	0.06	5.75	0.29	73.7	5.17	26.3	5.14	6.1	4.82	93.9	4.82	0.065	1.3	5.25	2.3	4.85	16.6	5.13	9.8	4.93	0.9	5.01	1.6	4.91	7.5	5.14	33.5	5.25	26.5	5.1
G+C15	3C15	16.05	1.01	0.05	6.31	0.31	67.5	5.05	32.5	5.16	1.0	5.15	99.0	5.15	0.010	0.6	4.9	0.0	5.05	24.2	5.24	11.1	5.15	0.2	5.15	0.2	5.11	7.5	4.90	13.3	4.96	42.9	5.19
G+C15	R3C15	20.40	1.37	0.07	6.69	0.34	73.4	5.02	26.6	5.13	8.1	5.34	91.9	5.34	0.088	1.1	5.10	5.0	5.17	16.4	5.12	9.8	4.94	1.1	5	0.9	5.19	8.0	5.25	35.7	4.96	22.0	5.24
G+C17	1C17	20.40	1.12	0.06	5.49	0.29	72.3	5.32	27.7	5.10	2.2	5.09	97.8	5.09	0.022	0.4	5.07	0.6	5.05	21.4	5.25	11.0	5.07	0.4	5.15	0.8	4.99	5.5	5.16	17.5	4.86	42.4	5.1
G+C17	R1C17	21.30	1.29	0.07	6.05	0.30	73.8	5.30	26.2	5.12	10.6	5.07	89.4	5.07	0.119	0.9	5.03	1.2	5.06	14.6	5.11	9.2	4.95	4.2	5.25	4.3	5.09	6.5	5.24	38.2	5.14	20.9	5.15
G+C17	3C17	23.70	1.16	0.06	4.88	0.25	73.5	5.34	26.5	4.97	3.6	5.33	96.4	5.33	0.037	0.5	4.92	2.0	4.95	19.9	5.23	10.7	5.10	0.4	5.18	0.7	5.01	5.7	4.95	18.3	4.97	41.8	5.01
G+C17	R3C17	21.15	1.40	0.07	6.60	0.34	73.1	5.15	26.9	5.16	13.4	5.33	86.6	5.33	0.155	0.6	4.88	0.8	5.12	15.9	5.17	8.9	4.87	5.1	5.21	6.9	5.17	5.3	5.24	32.1	5.05	24.4	4.97
C15	1C15	13.65	0.07	0.00	0.50	0.03	73.6	5.14	26.4	5.24	5.5	4.86	94.5	4.86	0.058	1.2	5.22	1.7	5.15	16.7	4.91	10.0	5.14	1.0	5.18	1.6	5.21	7.5	5.19	33.8	4.91	26.5	5.16
C15	R1C15	14.70	0.09	0.00	0.58	0.03	69.4	5.11	30.6	5.11	11.9	5.01	88.1	5.01	0.135	7.3	5.18	2.1	4.82	21.2	5.21	10.9	5.2	1.0	5.17	1.5	4.88	1.1	5.23	15.3	5.25	39.6	5.23
C15	3C15	13.50	0.08	0.00	0.57	0.03	73.6	5.07	26.4	5.07	7.3	5.18	92.7	5.18	0.079	0.7	5.28	4.5	5.11	16.6	5.24	9.9	5.29	1.1	5.16	1.0	5.01	8.0	4.89	35.9	5.13	22.3	5.12
C15	R3C15	15.15	0.10	0.01	0.64	0.03	65.4	5.15	34.6	5.15	18.8	5.21	81.2	5.21	0.232	11.1	5.31	4.6	4.8	21.3	5.24	9.7	4.81	1.4	5.07	1.7	5.06	0.8	5.11	11.7	5.22	37.7	4.92
C17	1C17	15.30	0.09	0.00	0.59	0.03	76.4	5.06	23.6	5.21	5.5	5.12	94.5	5.12	0.058	0.6	5.22	2.9	4.94	15.4	4.93	9.7	5.22	0.7	5.14	1.3	4.86	6.9	5.20	40.3	4.92	22.2	5.24
C17	R1C17	15.00	0.11	0.01	0.73	0.04	72.8	5.09	27.2	5.09	27.8	5.35	72.2	5.35	0.385	0.7	5.15	0.6	5.25	16.6	5.24	8.5	5.31	9.6	5.02	16.9	4.96	0.3	5.18	13.6	4.92	33.2	4.99
C17	3C17	18.45	0.14	0.01	0.78	0.04	73.2	4.89	26.8	4.93	12.5	5.19	87.5	5.19	0.143	1.0	4.82	1.3	4.82	16.0	5.16	9.0	4.96	4.4	4.94	5.8	5.18	5.4	4.88	32.5	5.11	24.6	5.14
C17	R3C17	16.95	0.14	0.01	0.81	0.04	70.2	5.01	29.8	5.15	33.5	5.07	66.5	5.07	0.504	0.7	4.85	0.9	4.93	14.5	5.22	7.8	4.91	14.4	4.9	17.5	5.03	0.2	5.17	13.4	4.91	30.6	5.19
Trichosporon cutaneum																																			
S	CH	L/X (% w/w)	L (g/L)	S.D.	X (g/L)	S.D.	unsat FA	S.D. (%)	sat FA	S.D. (%)	odd FA	S.D. (%)	even FA	S.D. (%)	odd/even ratio	15:0	S.D. (%)	15:1	S.D. (%)	16:0	S.D. (%)	16:1	S.D. (%)	17:0	S.D. (%)	17:1	S.D. (%)	18:0	S.D. (%)	18:1	S.D. (%)	18:2	S.D. (%)
G+C15	1C15	31.80	0.58	0.03	1.83	0.09	84.0	5.21	16.0	5.08	0.9	5.16	99.1	5.16	0.009	0.2	5.20	0.3	5.17	13.8	5.13	2.5	5.09	0.1	5.19	0.3	4.96	1.9	5.15	29.4	5.19	51.5	4.88
G+C15	R1C15	33.40	1.50	0.07	4.49	0.24	83.6	5.03	16.4	5.21	1.3	5.15	98.7	5.15	0.013	0.3	5.21	0.5	4.92	14.1	4.89	2.6	4.92	0.2	4.88	0.3	5.17	1.8	5.06	27.5	5.09	52.7	5.25
G+C15	3C15	30.90	0.60	0.03	1.94	0.10	82.9	4.91	17.1	5.1	1.2	5.00	98.8	5.00	0.012	0.2	5.07	0.4	5.02	14.7	5.18	2.1	5.14	0.2	5.07	0.4	4.92	2.0	5.01	26.7	4.91	53.3	5.26
G+C15	R3C15	32.80	2.37	0.12	7.22	0.36	83.6	4.99	16.4	5.01	1.9	5.06	98.1	5.06	0.019	0.4	4.93	0.8	4.95	13.8	5.07	2.8	4.91	0.3	4.98	0.4	5.24	1.9	4.87	28.4	5.06	51.2	5.09
G+C17	1C17	32.70	0.76	0.04	2.32	0.12	83.8	4.93	16.2	5.24	0.9	4.88	99.1	4.88	0.009	0.0	5.24	0.0	5.26	14.2	5.00	2.8	5.25	0.5	4.91	0.4	4.91	1.5	5.34	28.1	5.09	52.5	4.85
G+C17	R1C17	31.60	1.44	0.07	4.56	0.24	82.0	4.87	18.0	4.99	0.9	5.04	99.1	5.04	0.009	0.0	5.15	0.1	5.23	15.7	5.19	2.2	5.01	0.3	5.1	0.5	5.02	2.0	5.11	28.6	5.07	50.6	5.22
G+C17	3C17	31.80	0.70	0.04	2.19	0.11	82.8	5.07	17.2	5.18	1.0	5.13	99.0	5.13	0.010	0.0	5.12	0.1	4.89	15.3	5.17	2.9	4.93	0.3	4.85	0.6	4.98	1.6	5.18	26.4	5.12	52.8	5.14
G+C17	R3C17	30.70	1.59	0.08	5.19	0.27	83.4	4.95	16.6	5.22	1.4	5.24	98.6	5.24	0.014	0.0	5.01	0.2	4.86	14.6	5.21	2.3	5.13	0.5	5.14	0.7	5.03	1.5	5.25	28.1	4.85	52.1	5.25
C15	1C15	29.20	0.02	0.00	0.07	0.00	84.2	4.86	15.8	5.19	1.4	5.16	98.6	5.16	0.014	0.4	5.12	0.7	5.16	14.0	5.08	2.2	4.93	0.2	5.1	0.1	4.95	1.2	4.88	23.3	5.07	57.9	5.16
C15	R1C15	23.20	0.04	0.00	0.19	0.01	77.1	4.87	22.9	5.16	18.3	4.91	81.7	4.91	0.224	4.1	4.85	7.3	5.25	13.9	5.12	3.0	4.96	3.2	5.1	3.7	5.23	1.7	5.12	20.4	4.99	42.7	5.07
C15	3C15	28.30	0.02	0.00	0.06	0.00	83.3	5.04	16.7	5.04	2.6	5.03	97.4	5.03	0.027	0.8	5.21	1.3	5.11	14.2	5.08	2.0	5.04	0.3	5.03	0.2	5.17	1.4	5.15	24.1	5.02	55.7	5.02
C15	R3C15	23.10	0.07	0.00	0.30	0.02	73.8	4.95	26.2	5.14	27.4	5.17	72.6	5.17	0.377	7.2	4.94	9.8	4.97	12.3	5.2	2.4	5.09	4.9	5.04	5.5	5.18	1.8	5.16	16.8	4.99	39.3	5.11
C17	1C17	27.60	0.02	0.00	0.06	0.00	82.7	4.98	17.3	5.18	1.5	5.19	98.5	5.19	0.015	0.0	4.94	0.1	5.13	14.8	5.12	2.1	4.89	0.8	5.1	0.6	4.95	1.7	5.16	29.1	5.18	50.8	5.15
C17	R1C17	24.50	0.06	0.00	0.25	0.01	76.4	4.93	23.6	5.2	20.0	5.21	80.0	5.21	0.250	0.9	4.90	0.7	4.89	14.5	5.21	2.5	4.95	7.0	5.04	11.4	4.91	1.2	4.88	21.3	5.25	40.5	5.24
C17	3C17	25.40	0.02	0.00	0.07	0.00	81.9	4.91	18.1	4.89	2.3	5.13	97.7	5.13	0.024	0.1	4.96	0.2	5.18	15.1	4.85	2.0	5.1	1.1	5.01	0.9	5.09	1.8	5.09	27.2	5.21	51.6	4.93
C17	R3C17	23.60	0.03	0.00	0.13	0.01	75.5	4.95	24.5	4.9	26.5	5.08	73.5	5.08	0.361	1.2	5.23	0.9	5.03	12.5	5.15	2.2	4.87	9.5	5.03	14.9	5.09	1.3	4.94	17.9	4.93	39.6	5.05
Yarrowia lipolytica																																			
S	CH	L/X (% w/w)	L (g/L)	S.D.	X (g/L)	S.D.	unsat FA	S.D. (%)	sat FA	S.D. (%)	odd FA	S.D. (%)	even FA	S.D. (%)	odd/even ratio	15:0	S.D. (%)	15:1	S.D. (%)	16:0	S.D. (%)	16:1	S.D. (%)	17:0	S.D. (%)	17:1	S.D. (%)	18:0	S.D. (%)	18:1	S.D. (%)	18:2	S.D. (%)
G+C15	1C15	22.80	0.79	0.04	3.46	0.17	86.8	4.99	13.2	5.15	0.6	5.13	99.4	5.13	0.006	0.1	4.91	0.2	5.08	12.3	5.18	13.2	5.23	0.1	5.04	0.2	4.96	0.7	4.99	55.6	5.02	17.6	5.15
G+C15	R1C15	25.60	1.32	0.07	5.15	0.26	85.7	5.22	14.3	4.93	0.5	5.23	99.5	5.23	0.005	0.0	5.02	0.2	4.97	13.2	5.07	12.9	5.18	0.1	5.17	0.2	4.94	1.0	5.25	55.6	4.92	16.8	4.92
G+C15	3C15	21.90	1.11	0.06	5.08	0.25	84.9	5.24	15.1	5.19	0.5	5.09	99.5	5.09	0.005	0.1	5.14	0.2	5.14	13.8	4.86	11.9	4.91	0.0	5.12	0.2	5.17	1.2	5.22	54.7	5.02	17.9	5.22
G+C15	R3C15	24.80	1.43	0.07	5.78	0.28	86.1	5.03	13.9	5.09	0.5	5.13	99.5	5.13	0.005	0.1	5.08	0.3	4.87	12.6	5.2	13.7	4.97	0.0	4.89	0.1	4.93	1.2	4.9	56.8	4.91	15.2	5.01
G+C17	1C17	21.60	0.89	0.05	4.11	0.20	85.8	4.90	14.2	5.03	0.6	5.19	99.4	5.19	0.006	0.1	5.04	0.3	4.94	13.0	4.87	12.7	5.07	0.1	5.21	0.1	5.17	1.0	5.18	54.9	4.99	17.8	4.85
G+C17	R1C17	24.10	1.26	0.06	5.21	0.27	84.8	4.86	15.2	4.98	0.5	5.09	99.5	5.09	0.005	0.0	5.2	0.0	4.88	14.0	4.87	14.1	5.21	0.2	4.95	0.3	4.9	1.0	5.14	53.1	5.12	17.3	4.89
G+C17	3C17	20.10	0.84	0.04	4.19	0.21	86.0	5.15	14.0	5.11	1.0	4.9	99.0	4.9	0.010	0.2	5.22	0.4	4.91	12.6	5.04	13.5	5.16	0.1	5.19	0.3	5.13	1.1	5.17	54.5	4.93	17.3	4.91
G+C17	R3C17	23.30	1.24	0.06	5.31	0.27	85.5	4.91	14.5	5.08	0.4	4.87	99.6	4.87	0.004	0.0	4.92	0.1	5.21	13.3	4.92	14.0	4.85	0.1	5.14	0.2	4.99	1.1	4.87	56.4	5.24	14.8	5.11
C15	1C15	13.40	0.11	0.01	0.80	0.04	88.1	5.23	11.9	4.99	0.7	4.94	99.3	4.94	0.007	0.2	5.16	0.4	5.02	10.6	5.21	15.7	4.9	0.0	5.02	0.1	5.05	1.1	4.91	56.1	5.09	15.8	5.16

Table continues on next page

		Yarrowia lipolytica																															
S	CH	L/X (% w/w)	L (g/L)	S.D.	X (g/L)	S.D.	unsat FA	S.D. (%)	sat FA	S.D. (%)	odd FA	S.D. (%)	even FA	S.D. (%)	odd/even ratio	15:0	S.D. (%)	15:1	S.D. (%)	16:0	S.D. (%)	16:1	S.D. (%)	17:0	S.D. (%)	17:1	S.D. (%)	18:0	S.D. (%)	18:1	S.D. (%)	18:2	S.D. (%)
C15	R1C15	17.50	0.16	0.01	0.89	0.04	69.5	4.91	30.5	5.14	33.8	4.9	66.2	4.9	0.511	11.3	5.25	14.5	4.88	14.2	5.13	14.2	5.24	4.1	5.05	3.9	5.07	0.9	4.89	28.4	5.07	8.5	4.96
C15	3C15	13.50	0.37	0.02	2.73	0.13	87.8	5.10	12.2	5.07	1.3	4.95	98.7	4.95	0.013	0.4	4.93	0.7	5.12	10.8	4.86	14.9	4.96	0.1	5.25	0.1	5.12	0.9	4.99	52.7	5.24	19.4	4.98
C15	R3C15	17.20	0.47	0.02	2.75	0.14	67.4	5.16	32.6	5	44.5	4.94	55.5	4.94	0.802	15.6	4.98	19.8	5.05	11.7	5.04	10.8	5.19	4.6	5.05	4.5	5.01	0.7	4.97	24.7	5.02	7.6	5.21
C17	1C17	12.80	0.10	0.00	0.74	0.04	87.0	4.92	13.0	5.17	0.4	5	99.6	5	0.004	0.0	4.87	0.1	5.03	12.1	5.07	10.8	5.11	0.1	4.99	0.2	5.08	0.8	4.93	60.0	5.06	15.9	5.17
C17	R1C17	17.20	0.13	0.01	0.74	0.04	75.5	4.89	24.5	4.97	25.2	5.1	74.8	5.1	0.337	0.4	5.21	0.2	5.12	12.9	4.93	13.0	4.99	10.4	5.17	14.2	4.97	0.8	5.14	40.9	5.02	7.2	5.04
C17	3C17	12.40	0.34	0.02	2.72	0.14	87.1	5.18	12.9	4.98	0.7	4.86	99.3	4.86	0.007	0.1	4.9	0.2	5.19	11.7	5.12	12.3	4.93	0.1	5.14	0.3	5.01	1.0	4.97	57.5	4.97	16.8	5.04
C17	R3C17	17.00	0.46	0.02	2.73	0.14	73.3	5.11	26.7	4.89	31.3	4.99	68.7	4.99	0.456	0.5	4.97	0.5	5.13	13.7	4.94	12.5	4.89	12.4	5.05	17.9	5.02	0.1	5.08	34.5	4.88	7.9	5.17

Fig. 1S Growth curves of *T. cutaneum* on glucose with pentadecane (A) and glucose with heptadecane (B) as a carbon source (R = addition of rhamnolipid).

A

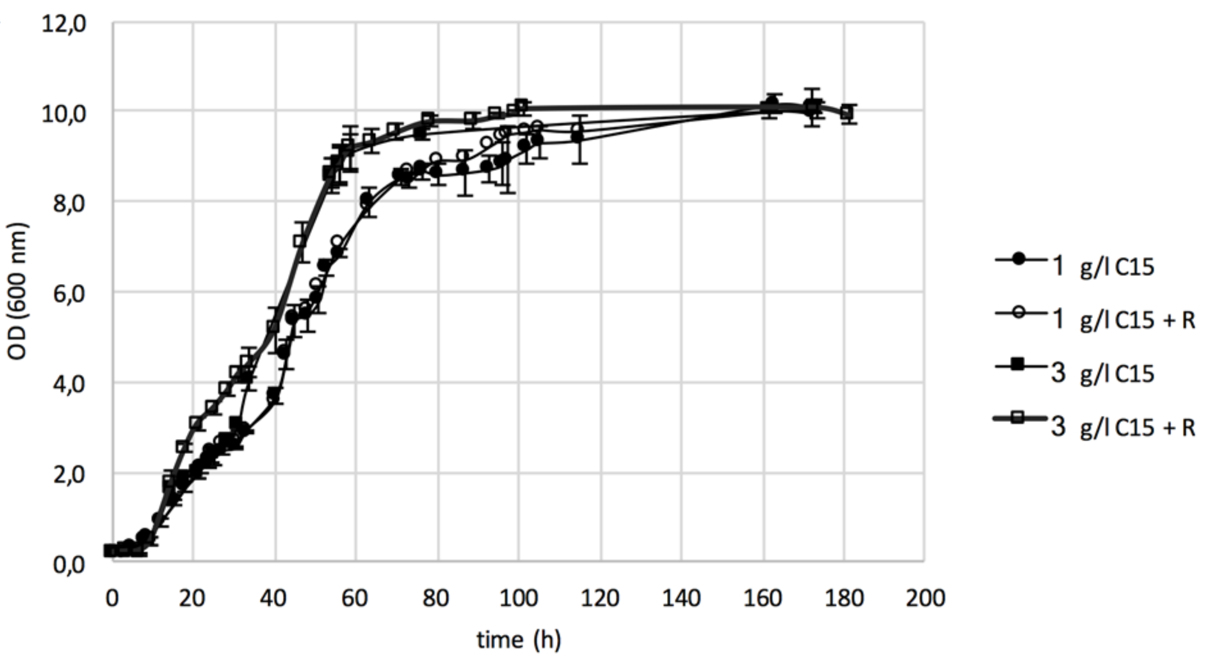

B

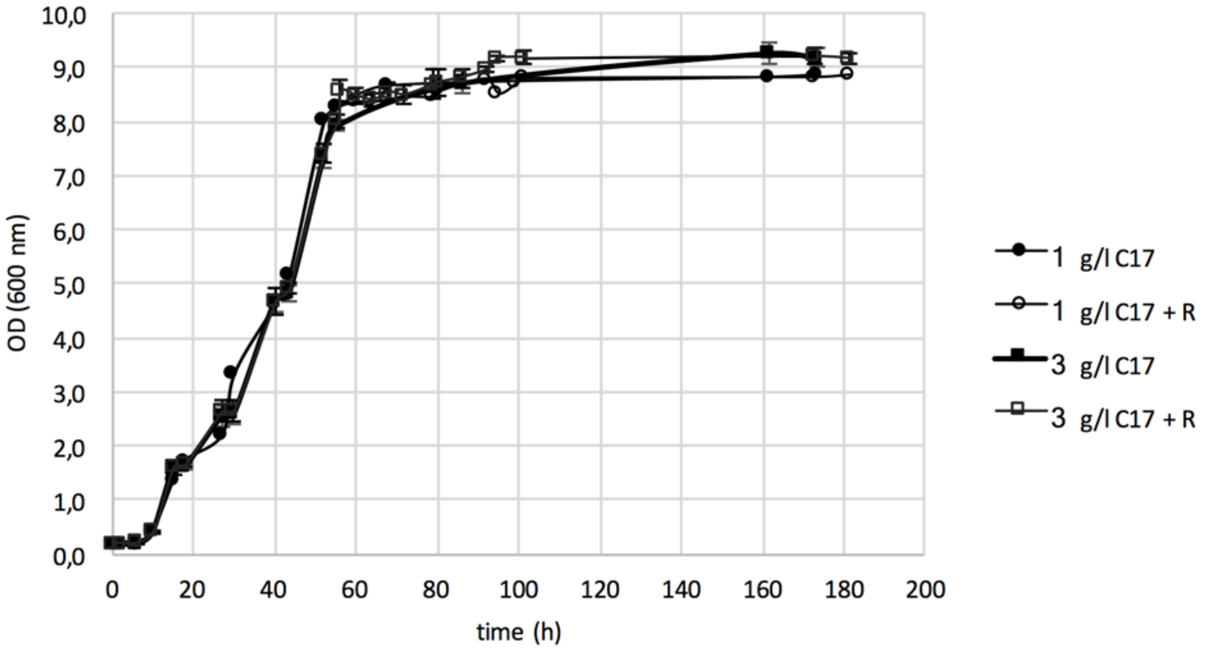

Fig. 2S Growth curves of *T. cutaneum* on pentadecane (A) and heptadecane (B) as a sole carbon source (R = addition of rhamnolipid).

A

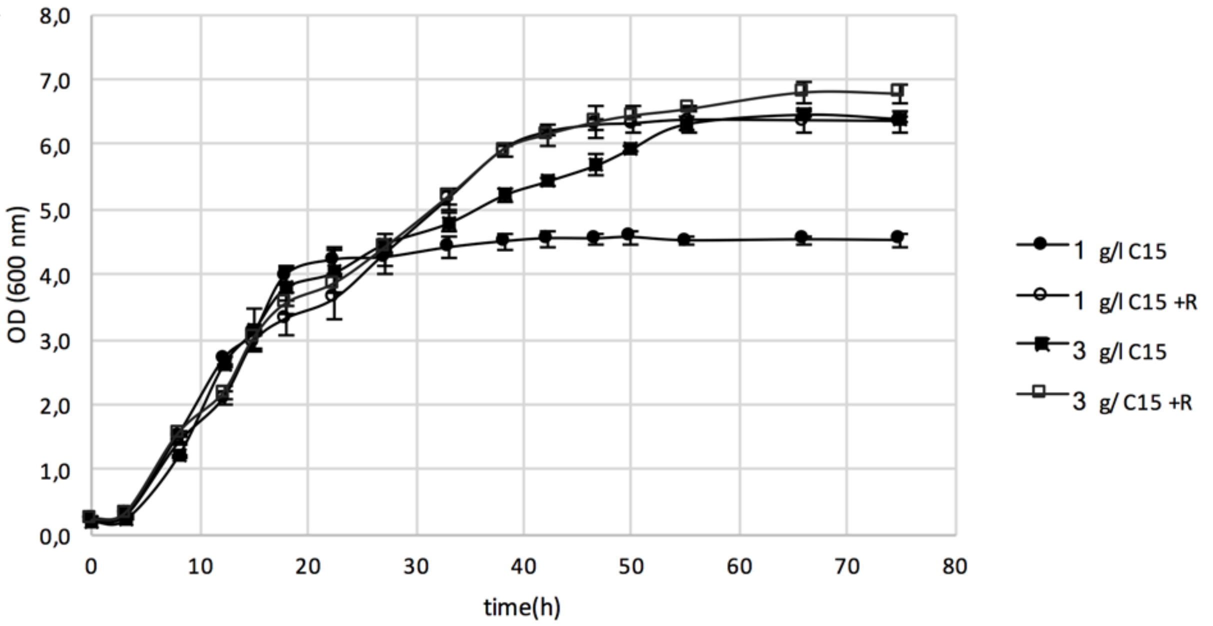

B

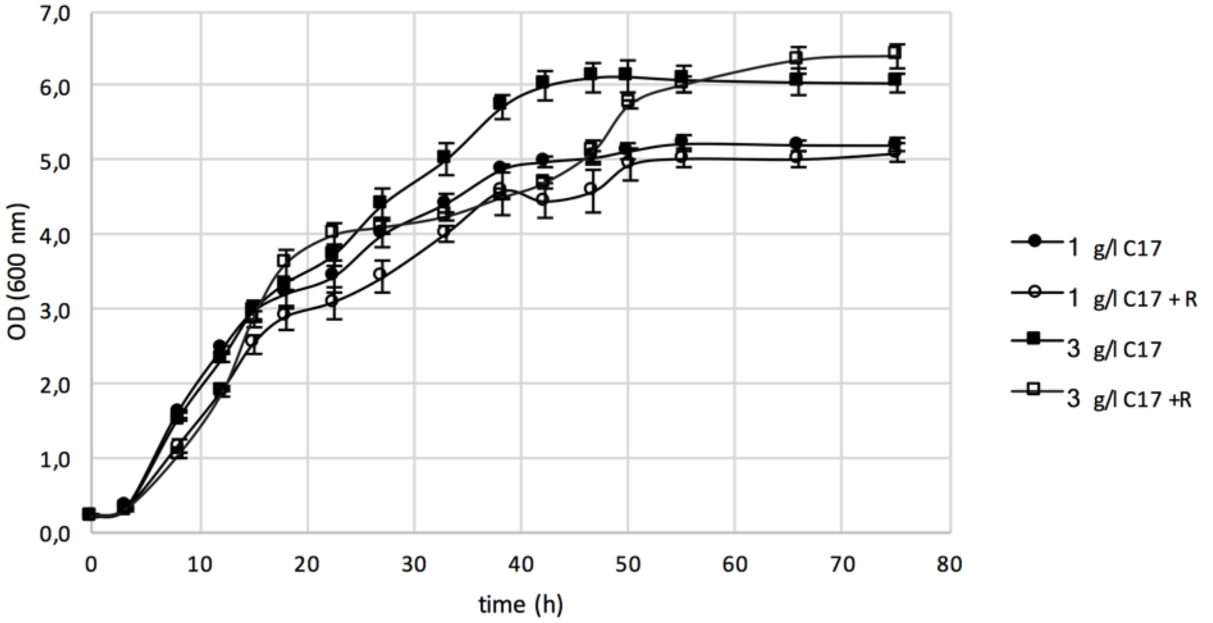

Fig. 3S Growth curves of *C. krusei* on glucose with pentadecane (A) and glucose with heptadecane (B) as a carbon source (R = addition of rhamnolipid).

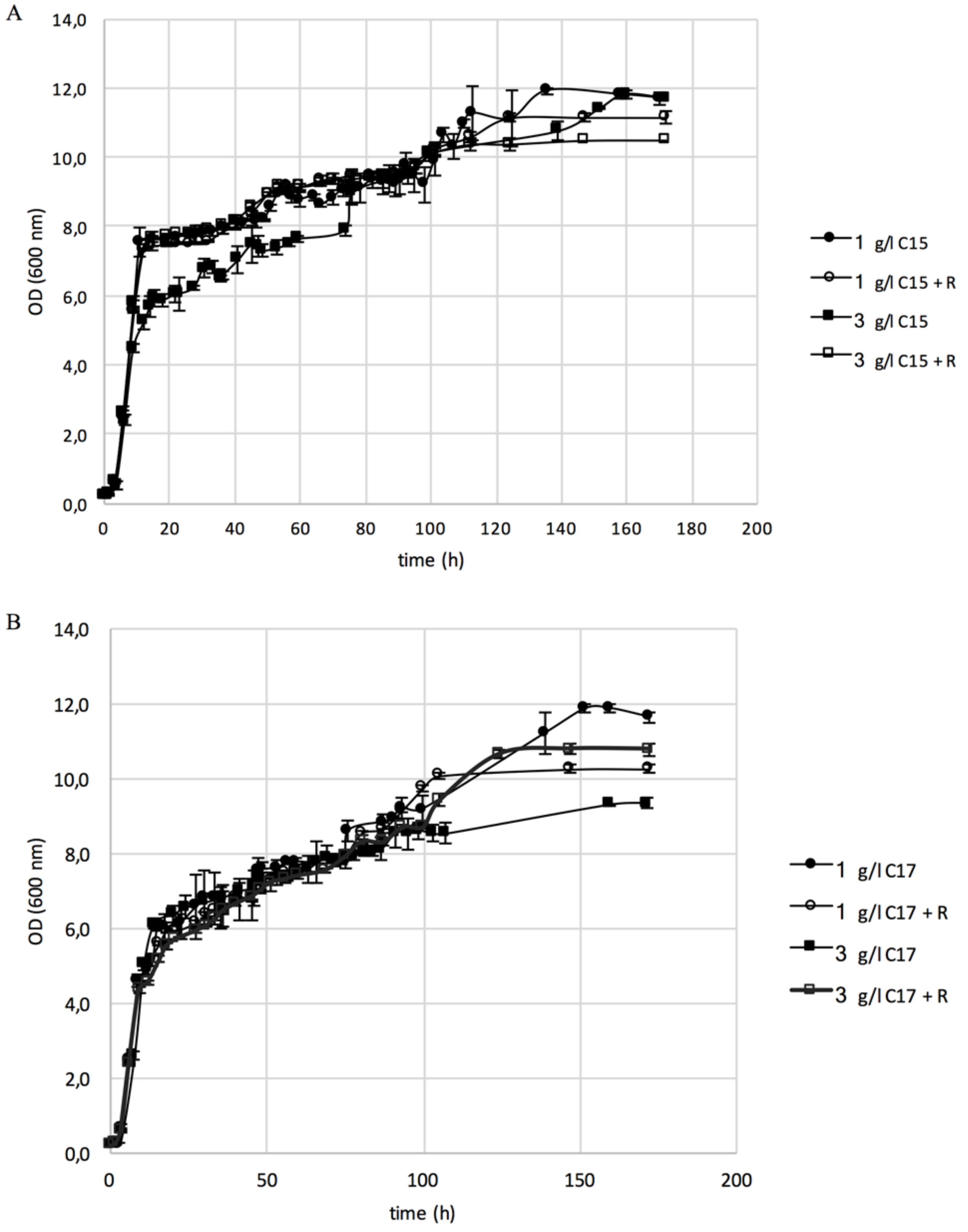

Fig. 4S Growth curves of *C. krusei* on pentadecane (A) and heptadecane (B) as a sole carbon source (R = addition of rhamnolipid).

A

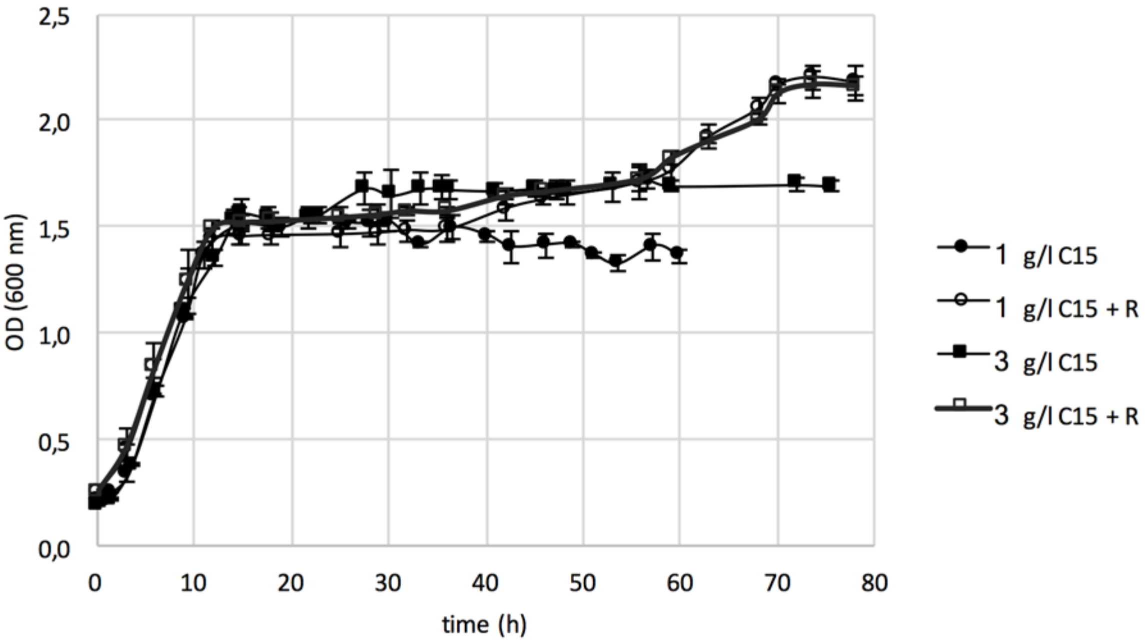

B

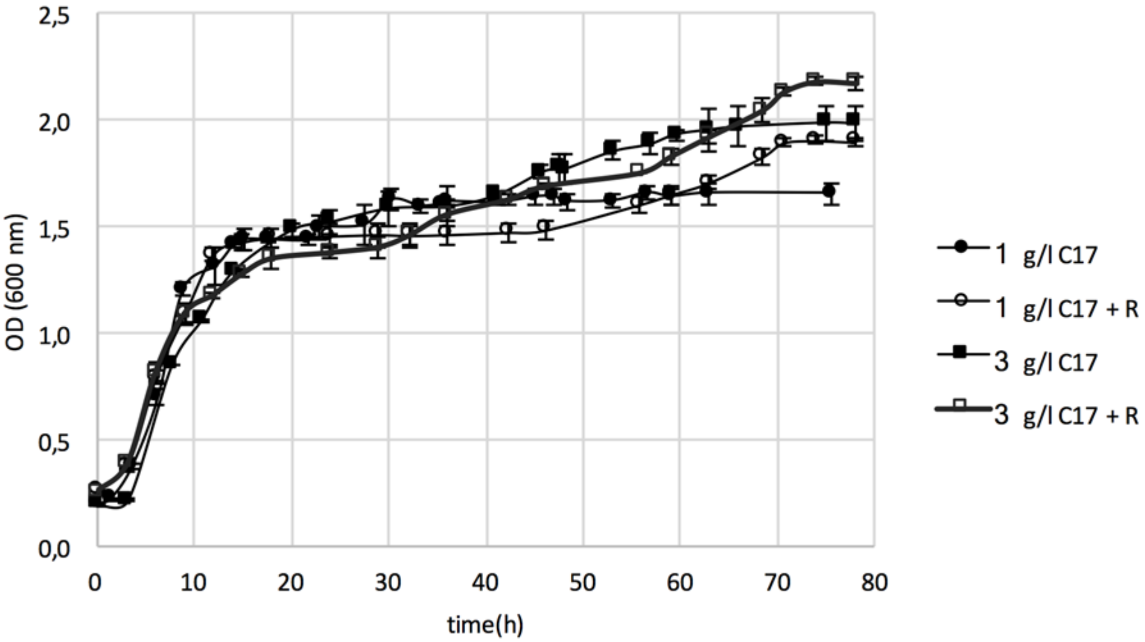

Supplement: Supplementary file 1 — Effect of different substrates (pentadecane C15; heptadecane C17 in concentration 1 g/L or 3 g/L) with or without presence of rhamnolipid (R) or glucose (G) on biomass yield (X), total lipid production (L) and lipid yield (L/X), and the proportion of saturated, unsaturated, odd, and even fatty acids by three yeast strains. The highest odd fatty acid content is shown in bold. The standard deviations are included. [file 8195329.f1.pdf]
